# A literature review of applied adaptive design methodology within the field of oncology in randomised controlled trials and a proposed extension to the CONSORT guidelines

**DOI:** 10.1186/s12874-017-0393-6

**Published:** 2017-07-18

**Authors:** Pankaj Mistry, Janet A Dunn, Andrea Marshall

**Affiliations:** Warwick Clinical Trials Unit, University of Warwick, Gibbet Hill Road, Coventry, CV4 7AL UK

**Keywords:** Adaptive, Adaptive design, Review, Clinical trials, Cancer, Interim analysis

## Abstract

**Background:**

The application of adaptive design methodology within a clinical trial setting is becoming increasingly popular. However the application of these methods within trials is not being reported as adaptive designs hence making it more difficult to capture the emerging use of these designs. Within this review, we aim to understand how adaptive design methodology is being reported, whether these methods are explicitly stated as an ‘adaptive design’ or if it has to be inferred and to identify whether these methods are applied prospectively or concurrently.

**Methods:**

Three databases; Embase, Ovid and PubMed were chosen to conduct the literature search. The inclusion criteria for the review were phase II, phase III and phase II/III randomised controlled trials within the field of Oncology that published trial results in 2015. A variety of search terms related to adaptive designs were used.

**Results:**

A total of 734 results were identified, after screening 54 were eligible. Adaptive designs were more commonly applied in phase III confirmatory trials. The majority of the papers performed an interim analysis, which included some sort of stopping criteria. Additionally only two papers explicitly stated the term ‘adaptive design’ and therefore for most of the papers, it had to be inferred that adaptive methods was applied. Sixty-five applications of adaptive design methods were applied, from which the most common method was an adaptation using group sequential methods.

**Conclusions:**

This review indicated that the reporting of adaptive design methodology within clinical trials needs improving. The proposed extension to the current CONSORT 2010 guidelines could help capture adaptive design methods. Furthermore provide an essential aid to those involved with clinical trials.

**Electronic supplementary material:**

The online version of this article (doi:10.1186/s12874-017-0393-6) contains supplementary material, which is available to authorized users.

## Background

In recent years, there has been a rise of interest in adaptive design methodology [1, 2]. The Food and Drug Administration (FDA) define an adaptive design as “a study that includes a prospectively planned opportunity for modification of one or more specified aspects of the study design and hypotheses based on analysis of data from subjects in the study” [3]. Conversely Chow and Chang (2008) have broadened this definition by classifying adaptive designs as any modifications made prospectively, concurrently or retrospectively during the conduct of a trial.

The implementation of adaptive designs can have potential benefits in clinical trials over other study designs such as parallel design, crossover etc. [4]. They can be more efficient, more cost effective, the likelihood of success increases, and there is an improved understanding of treatment success [1, 4, 5], however these benefits are only possible if the validity and integrity of the proposed study isn’t undermined [1]. Furthermore adaptive design methods may appeal more to clinical investigators due to the flexibility and prospect of making changes based on data at an interim stage of the trial.

Previous reviews into the application of adaptive design methodology have already been conducted. For example, Bauer (2006) conducted a review to investigate papers that were published between the years 1989 and 2004. The purpose of their review was to explore the impact of adaptive design methodology in medicine, particularly those designs based on the combination test or conditional error function, and to see if these methods were applied and presented appropriately [6]. A review conducted by Stevely (2015) assessed the standards of phase III group sequential randomised controlled trials (RCT) against the CONSORT 2010 checklist [7]. This review looked at papers published between 1st January 2001 and 23rd September 2014. Eligible papers were found in 11 different therapeutic fields, of which the majority (76%) were found in the field of oncology. This review concluded that there were issues with the reporting of group sequential trials and hence suggest an extension to the CONSORT checklist to help solve this problem. A recently published review by Hatfield (2016) examines the state of phase II, II/III and III adaptive design trials registered between the years 2000 and 2014. The ClinicalTrials.gov website and National Institute for Health Research register were used to collate registered trials with adaptive designs. Of all the registered trials with adaptive designs, the review found that adaptive designs are most often used in the field of oncology. The review did not successfully capture all trials with adaptive designs, and hence suggested that clinical trial registers should dedicate sections for adaptive designs and encourage the use of the term ‘adaptive design’ in either the title, summary or design sections of the register [8].

The aforementioned reviews have looked into specific methods related to adaptive design methodology or attempted to find all adaptive designs registered on Clinical trial registries. It is unclear the current extent of applications of adaptive design methodology specifically in oncology trials and whether reporting has improved over time. Therefore this literature review specifically attempts to capture papers published in 2015 that are using adaptive design methodology within phase II, II/III or III RCT in the field of Oncology. This review aims to understand how adaptive design methodology is currently being reported within journals, whether these methods are being applied prospectively or concurrently, what methods are commonly applied and to understand whether the use of adaptive design methodology is being explicitly stated or whether it has to be inferred. Furthermore this review will suggest extensions to the CONSORT that can be implemented to aid better reporting and to maximise the capture of adaptive design methodology.

## Methods

### Literature search

The Embase, Ovid and PubMed databases were chosen to conduct the literature search into the application of adaptive design methodology. The review was constrained to phase II, phase III or phase II/III RCT’s with patients diagnosed with cancer that presented primary outcome trial results and were published in 2015. Eligible papers should indicate the use of adaptive design methodology, papers should be full text publications in the English language and accessible. Duplicate records were excluded based on the title, authors, abstract and year of publication. All required data were extracted and recorded on an excel spreadsheet.

The definition that will be used to identify the application of adaptive design methodology will be any potential modifications made to the trial/statistical procedure that is either prospective, ad-hoc or retrospective [1, 9].

A free text search was conducted to capture phase II, II/III, or IIII Cancer RCTs, the following keywords were used: “phase II(2)”, “phase III(3)”, “phase II(2)/III(3)”, “Oncology”, “Cancer”, “Neoplasm”, “Carcinoma”, “randomis(z)ed controlled trial(s)”, “randomis(z)ed clinical trial(s)”, “trial”, “controlled clinical trial(s)”. To capture as many results related to adaptive design methodology within these trials, the following keywords were used alongside the Boolean operator “OR”: “adaptive design(s)”, “flexible design(s)”, “group sequential”, “adaptive randomis(z)ation”, “sample size re-estimation”, “sample size adjustment”, “sample size modification”, “MAMS”, “multi(−)arm multi(−)stage”, “multi(ple) arm”, “multi(ple) stage”, “interim analysi(e)s”, “adaptive seamless”, “biomarker adaptive”, “adaptive clinical trial(s)”, “two-stage adaptive”, “multiple adaptive”, “adaptive enrichment”, “dose escalation”, “dose selection”, “drop the loser”, “pick the winner” and “treatment switch(ing)”.

### Data extraction

An excel spreadsheet was used to record the following data:

- Standard demographics such as first author, title, name of the trial;
- The journal that the paper was published in;
- The funder or sponsor of the study;
- The phase of the trial;
- The type of cancer being reported;
- The nature of the primary outcome;
- The number of trial arms;
- The type of intervention being implemented;
- The number of any planned interim analyses;
- The stage of the trial being reported, i.e. interim or final analysis;
- Any planned stopping criteria and reason;
- Whether the trial was terminated early and the reason for early stopping;
- The initial planned sample size and the reported sample size;
- The adaptive designs methodology used;
- The number of adaptive designs that were applied;
- Whether the adaptive design was predetermined or concurrent;
- Whether the use of adaptive designs was explicitly stated;
- The trial identifier if it had been registered on clinical trial websites.

The papers identified by the literature search were all reviewed and information extracted and recorded in the aforementioned excel spreadsheet. Data that could not be found in the paper was researched by using the trial identifier or trial name to find out the relevant information. If no further information was available then the data was classified as missing. One person (PM) extracted the information from the papers and any papers that needed further clarification were checked and validated by two reviewers as a form of quality control (AM and JD).

## Results

**Fig. 1:**
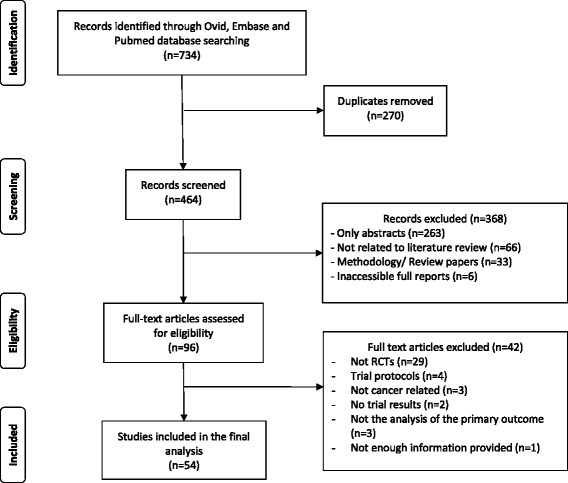
Modified PRIMSA flow diagram showing the review process

A total of 8288 records were identified related to RCTs in the field of Oncology that were published in 2015 across the Ovid, Embase and PubMed databases. Of which 734 records were identified using the key search terms mentioned above that were related to adaptive designs within the phase II, phase II/III and phase III trial setting.. After the removal of duplicates 464 records were screened, of which 368 records were excluded due to the following reasons: published abstract only (*n* = 263), not related to the literature review (*n* = 66), either methodology or review papers hence would not contain results (*n* = 33) or the full reports were inaccessible (*n* = 6). The remaining 96 full text records were further assessed for eligibility, of which 42 were excluded for the following reasons: not a RCT (*n* = 29), not cancer related (*n* = 3), not the analysis of the primary outcome (*n* = 9) or no information provided (*n* = 1). This left a total of 54 records for analysis (Fig. 1) (see Additional file 1 for the full list of titles for each paper).

Of the 54 papers reviewed, 38 papers (70%) were phase III confirmatory trials, 12 out of 54 papers (22%) were phase II trials, 2 (4%) were phase IIb trials and 2 (4%) were phase II/III trials. The number of arms in a trial ranged from two to five arms, 46 (85%) papers were two arm trials of which 35 of these were phase III trials, 10 of the two arm trials were phase II trials, there was 1 four arm phase III trial and 1 five arm phase II/III trial (Table 1).

**Table 1:** Two way table of phase of a trial and number of trial arms it has

Trial phase	Number of arms				Total
	2	3	4	5	
Phase II	10	2	0	0	12
Phase IIb	1	1	0	0	2
Phase II/III	0	1	0	1	2
Phase III	35	2	1	0	38
Total	46	6	1	1	54

The literature review identified adaptive trials being reported in 21 different journals (Appendix 1). Of these the Journal of Clinical Oncology published the highest number of papers with 12 out of 54 papers (22%), followed by the Lancet Oncology with 11 papers (21%), then the New England Journal of Medicine with 6 papers (11%) and the European Journal of Cancer publishing 3 papers (6%). The remaining 17 journals published either one or two papers (Appendix 1).

There were 45 out of 54 (81%) papers that had a time to event outcome as its primary outcome (Appendix 2). Furthermore 49 out of 54 (92%) were drug related trials (Appendix 3).

Of the 54 papers reviewed, 33 (61%) were published based on results during an interim analysis, the results of the remaining papers were based on either final analysis (20/54) or subgroup analysis (1/54). Of the 33 papers published based on results during an interim analysis, 26 papers resulted in the trial stopping early, of which all 26 had a pre-planned stopping criteria that stopped early due to either safety/efficacy/futility i.e. group sequential methods (Table 2). The remaining 7 papers stated to continue with the trial as planned. The majority of the papers (48/54) had a pre-planned interim analysis with 34 out of the 48 specifying one interim analysis during the course of the trial, 9 specifying two interim analyses, 3 specifying three interim analyses and the remaining 2 papers conducting interim analyses annually during the course of the trial.

**Table 2:** Two way table of whether the trial stopped early compared to if the trial had a planned stopping criteria

Trial stopped early	Pre-planned stopping criteria			Total
	No	Yes	Unknown	
No	5	22	1	28
Yes	0	26	0	26
Total	5	48	1	54

Majority of trials applied a single adaptive design method (44/54 papers, 80%), 9 out of 54 papers (19%) applied two methods and 1 paper (2%) applied three methods. In total there were 65 applications of adaptive design methods, of which the most reported method was adaptations using group sequential methods with 50 out of 65 applications, followed by dose modifications (8/65), sample size re-estimation (4/65), adaptive randomisation (1/65),) change in primary endpoint (1/65) and change in patient eligibility (1/65).

Table 3 shows the different variables extracted split by the adaptive method applied. All papers that applied group sequential methods had incorporated a planned stopping criteria. Furthermore 35/50 of these group sequential methods were in a phase III setting. All 4 papers that implemented sample size re-estimation methods resulted in the trial stopping early. Additionally 3 out of 4 of these papers had both pre-determined and ad-hoc applications of adaptive design methods. Conversely all papers that applied dose modification methods had pre-determined the use of adaptive design methods.

**Table 3:** The data extracted split by the adaptive method applied

Data extracted	Adaptive method applied					
	Group sequential methods (*n* = 50)	Dose modifications (*n* = 8)	Sample size re-estimation (*n* = 4)	Adaptive randomisation (*n* = 1)	Change in primary endpoint (*n* = 1)	Change in patient eligibility (*n* = 1)
Trial phase						
II	13	2	0	1	0	0
II/III	2	1	1	0	0	0
III	35	5	3	0	1	1
Number of arms						
2	42	7	2	1	1	1
3	6	1	0	0	0	0
4	1	0	1	0	0	0
5	1	0	1	0	0	0
Stage of reporting						
Interim analysis	33	2	2	0	1	1
Subgroup analysis	0	1	0	0	0	0
Final analysis	17	5	2	1	0	0
Planning of adaptive design method						
Pre-determined	45	8	1	1	0	1
Ad-hoc	1	0	0	0	0	0
Both	4	0	3	0	1	0
Explicitly stated Adaptive design						
Yes	1	1	1	1	0	0
No	49	7	3	0	1	1
Planned stopping criteria						
Yes	50	5	4	0	1	1
No	0	3	0	1	0	0
Trial stopped early						
Yes	26	1	4	0	1	0
No	24	7	0	1	0	1

Of the 54 papers reviewed, 49 (91%) had predetermined the use of adaptive design methods, 1 paper had applied ad-hoc adaptive design methods and 4 papers had predetermined and ad-hoc use of adaptive design methods. However only 2 out of 54 papers (4%) had explicitly used the phrase ‘adaptive design’ to explain that adaptive methodology was applied, the remaining papers indicated the use of adaptive design methods. Of the papers that explicitly stated the use of adaptive design methodology, one paper had implemented a Bayesian adaptive response design which included adaptive randomisation and dose modification. The other paper applied multiple adaptive methods which included the use of group sequential methods, sample size re-estimation, and dose modification.

## Discussion

This literature review aimed to understand the reporting of adaptive design methodology in RCTs in particular within the field of oncology. This review has highlighted that the reporting of these methods needs improving, which confirms the outcome of other reviews. Stevely et al. reported that there are issues related to the reporting of group sequential trials and suggested a consort extension to alleviate the issues related to this [7]. Hatfield et al. emphasises the need for improving the way adaptive designs are reported and suggests a modification to the CONSORT statement [8]. Additionally Bauer and Einfalt suggest that the presentation of adaptive design methodology needs to be developed [6].

The classifications of the adaptive design methods were based upon those mentioned by the FDA. One form of adaptive design methods are those using group sequential methods, these methods have been used extensively for a number of years hence the robustness of these methods have qualified them to be known as well understood methods by the regulators [3, 10]. This design employs stopping boundaries at regular interims to assist in decision making with regards to the trial or treatment. Many methods such as Simon’s two-stage design, O’Brien and Fleming design, multi-arm multi-stage designs can be included within the umbrella of group sequential designs [11–14]. Hence any papers that employed the aforementioned designs or applied methods whereby trial/treatment related decisions could be made during an interim analysis were classed as group sequential methods. It was found from the literature review that group sequential methods were used within 50 out of 65 applications.

The review found that majority of the papers (53/54) applied adaptive methods that were prospectively planned hence supporting the definition given by the FDA. The list of search terms helped in capturing applications of adaptive design methodology however only two papers explicitly stated the term ‘adaptive design’ and it was inferred from the remaining papers that adaptive design methodology was used.

The reporting of these studies has not improved and hence this review supports the need for a set of guidelines of how adaptive designs should be reported. A proposed extension to the current CONSORT 2010 guidelines has been made (Table 4). This extension should be used for any trials that fit the definition of adaptive designs as used for this literature review, i.e. for trials with any modifications made to the trial/statistical procedure that was either prospective, ad-hoc or retrospective. The justification for attempting to create an extension to the CONSORT guidelines is to ensure that as many adaptive design based trials are captured. A crucial question that needs to be answered is ‘at what point exactly a trial become classed as adaptive?’ Table 4 can assist that decision for those involved in the running of a trial.

**Table 4:** Proposed extensions to the current CONSORT diagram [15]

Section/Topic	Item No	Standard Checklist item	Extension for adaptive designs
Title and abstract			
	1a	Identification as a randomised trial in the title	Identification as an adaptive randomised trial if it is an adaptive design
	1b	Structured summary of trial design, methods, results, and conclusions (for specific guidance see CONSORT for abstracts [16, 17])	Include the term ‘adaptive design’ or ‘adaptive methods’
Introduction			
Background and objectives	2a	Scientific background and explanation of rationale	Rational for implementing an adaptive design
	2b	Specific objectives or hypotheses	
Methods			
Trial design	3a	Description of trial design (such as parallel, factorial) including allocation ratio	Define what adaptive design/ adaptive method will be applied
	3b	Important changes to methods after trial commencement (such as eligibility criteria), with reasons	Any changes during the trial should be reported as an adaptive method.
Participants	4a	Eligibility criteria for participants	Any changes in eligibility during the trial, should be classed as an adaptive design or adaptive method.
	4b	Settings and locations where the data were collected	
Interventions	5	The interventions for each group with sufficient details to allow replication, including how and when they were actually administered	
Outcomes	6a	Completely defined pre-specified primary and secondary outcome measures, including how and when they were assessed	
	6b	Any changes to trial outcomes after the trial commenced, with reasons	Any changes during the trial are classed as an adaptive method and should be mentioned.
Sample size	7a	How sample size was determined	Any changes to sample size or power during trial classed as an adaptive design or adaptive method and should be mentioned.
	7b	When applicable, explanation of any interim analyses and stopping guidelines	Explain why the interim analysis will be taking place, if potential pre-planned adaptations during interim analysis taking place then these should be mentioned in the methods as well (3b). Include details of any planned stopping boundaries for either the trial or dropping any of the intervention arms.
Randomisation:			
Sequence generation	8a	Method used to generate the random allocation sequence	
	8b	Type of randomisation; details of any restriction (such as blocking and block size)	Details if adaptive randomisation has been implemented.
Allocation concealment mechanism	9	Mechanism used to implement the random allocation sequence (such as sequentially numbered containers), describing any steps taken to conceal the sequence until interventions were assigned	
Implementation	10	Who generated the random allocation sequence, who enrolled participants, and who assigned participants to interventions	
Blinding	11a	If done, who was blinded after assignment to interventions (for example, participants, care providers, those assessing outcomes) and how	
	11b	If relevant, description of the similarity of interventions	
Statistical methods	12a	Statistical methods used to compare groups for primary and secondary outcomes	Details of how the adaptive design or the adaptive methods were applied Details of how the statistical methods were evaluated before implementation i.e. through the use of simulations?
	12b	Methods for additional analyses, such as subgroup analyses and adjusted analyses	
Results			
Participant flow (a diagram is strongly recommended)	13a	For each group, the numbers of participants who were randomly assigned, received intended treatment, and were analysed for the primary outcome	Ensure any adaptations are shown on this diagram, such as dropping of arms, treatment switching.
	13b	For each group, losses and exclusions after randomisation, together with reasons	
Recruitment	14a	Dates defining the periods of recruitment and follow-up	
	14b	Why the trial ended or was stopped	Any changes to recruitment during trial classed as an adaptive method, should be mentioned.
Baseline data	15	A table showing baseline demographic and clinical characteristics for each group	
Numbers analysed	16	For each group, number of participants (denominator) included in each analysis and whether the analysis was by original assigned groups	
Outcomes and estimation	17a	For each primary and secondary outcome, results for each group, and the estimated effect size and its precision (such as 95% confidence interval)	
	17b	For binary outcomes, presentation of both absolute and relative effect sizes is recommended	
Ancillary analyses	18	Results of any other analyses performed, including subgroup analyses and adjusted analyses, distinguishing pre-specified from exploratory	
Harms	19	All important harms or unintended effects in each group (for specific guidance see CONSORT for harms [18])	
Discussion			
Limitations	20	Trial limitations, addressing sources of potential bias, imprecision, and, if relevant, multiplicity of analyses	
Generalisability	21	Generalisability (external validity, applicability) of the trial findings	If ad-hoc adaptive methods were implemented, at what point was it decided to implement this and why.
Interpretation	22	Interpretation consistent with results, balancing benefits and harms, and considering other relevant evidence	
Registration	23	Registration number and name of trial registry	
Protocol	24	Where the full trial protocol can be accessed, if available	First and last protocol, with a list of amendments made.
Funding	25	Sources of funding and other support (such as supply of drugs), role of funders	

Pragmatically this extended CONSORT would greatly assist in efficient capturing of published papers related to adaptive design methodology. Furthermore extending the CONSORT 2010 guidelines would encourage greater capture of predetermined adaptations which would greatly benefit all those involved in clinical trials ensuring explicit and thorough reporting of the adaptive nature of RCTs and the methodology used. It will allow full transparency of all adaptations carried out during the trial.

## Conclusions

It can be concluded that the reporting of adaptive design methodology within RCT’s is inadequate and requires improvement. To assist in the capture of adaptive design methods the proposed extension to the CONSORT 2010 guidelines can be implemented; this will also prove to be a crucial aid to all members involved with clinical trials.

## Appendix 1

**Table 5:** List of journals from which the papers were published in

Journal	Frequency
Annals of Oncology	2
BMC Research Notes	1
Breast Cancer Research & Treatment	1
British Journal of Haematology	2
Cancer	2
Cancer Management and Research	1
Clinical Cancer Research	1
Clinical Lung Cancer	2
Drug design, development & therapy	1
European Urology	1
European Journal of Cancer	3
Gynaecologic Oncology	2
Head & Neck	1
Journal of American Medical Association	1
Journal of Clinical Oncology	12
Journal of Urology	1
Lancet Oncology	11
Molecular Cancer Therapeutics	1
New England Journal of Medicine	6
Leukemia	1
Radiotherapy & Oncology	1
Total	54

## Appendix 2

**Table 6:** List of primary outcomes of the papers

Primary outcome	Frequency
Bowel movement/Flushing episodes	1
Cytogenic Response	2
Disease Control Rate	1
Optimal dose	1
Overall pain response	1
Response to treatment	1
Success/Failure	2
Time to event outcome	45
Total	54

## Appendix 3

**Table 7:** List of categorised interventions of the papers

Intervention	Frequency
Adding acid	1
Drug	49
Surgery/Chemotherapy/Radiotherapy	3
Vaccine	1
Total	54

## Additional file

Additional file 1: Dose modification. (XLSX 14 kb) (XLSX 14 kb)

## Abbreviations

FDA: Food and Drug Administration
RCT: Randomised controlled trials
SAP: Statistical analysis plan
